# Not Just Another Deep Vein Thrombosis: Unmasking May-Thurner Syndrome

**DOI:** 10.7759/cureus.107835

**Published:** 2026-04-27

**Authors:** Muhammad Sohail Muhammadi, Aliza Zakaria, Aiman Balouch, Nadeem Ahmad, Suraj Puthentharayil Haridas

**Affiliations:** 1 Acute Medicine, Northern Lincolnshire and Goole NHS Foundation Trust, Scunthorpe, GBR; 2 Acute Internal Medicine, Northern Lincolnshire and Goole NHS Foundation Trust, Scunthorpe, GBR; 3 Acute Medicine, Scunthorpe General Hospital-Humber Health Partnership, Scunthorpe, GBR

**Keywords:** anticoagulation therapy, deep vein thrombosis (dvt), iliac vein compression, may-thurner syndrome (mts), rare

## Abstract

May-Thurner syndrome (MTS) is a condition characterised by the compression of the left iliac vein by the right common iliac artery, leading to venous stasis and an increased risk of deep vein thrombosis (DVT). This case report discusses a 56-year-old woman presenting with progressive swelling and pain in her left leg. Imaging studies revealed a significant left iliofemoral deep vein thrombosis and venous collateralization, leading to a diagnosis of May-Thurner syndrome. Despite the elevated levels of D-dimer and inflammatory markers in circulation, the thrombophilia screening revealed that she carried a heterozygous prothrombin 20210A allele, which contributes to her vulnerability to thrombotic events. The utilisation of Doppler ultrasound and CT venography was essential to validate the diagnosis, which is often challenging due to the nonspecific nature of the signs. The patient received anticoagulation therapy as part of a conservative treatment approach, which included careful monitoring and collaboration with various other specialties. This case underscores the importance of prompt identification of MTS of the left leg in patients presenting with leg swelling, pain, or deep vein thrombosis, especially in high-risk individuals, including those who have undergone surgery or have a familial predisposition. The primary focus of management involves anticoagulation, preventing recurrence, and effectively addressing complications.

## Introduction

May-Thurner syndrome (MTS) is a vascular anomaly characterized by the compression of the common iliac vein by the common iliac artery, usually the left common iliac vein by the right common iliac artery against the spine, most often at L5. The abnormal anatomy of the lower limb could contribute to poor venous drainage of the left lower limb, which would lead to a higher probability of development of deep vein thrombosis (DVT). Even though a lot of people who have MTS may not have the symptoms, some can have problems with their legs, discomfort, and chronic venous insufficiency.

The difficulty of diagnosing MTS is that the disorder has non-specific and frequently insidious symptoms that can express themselves in the form of swelling of the leg or the feeling of weight on the diseased limb. The result of this is that the condition is often overlooked or misdiagnosed and hence not treated, and there is a risk of developing complications such as DVT.

The case report will focus on providing a detailed description of a patient with a diagnosis of May-Thurner Syndrome, its clinical manifestation, diagnostic issues, and treatment plan. The present case demonstrates that diagnosing and treating MTS can be rather complicated, particularly when the patient has a number of risk factors that can distort the clinical picture.

This case is important because it highlights the importance of timely diagnosis of May-Thurner Syndrome in patients who present with leg swelling or pain, especially in cases where there is a possibility of deep vein thrombosis of the left leg. With early diagnosis and proper intervention, the severe complications can be reduced, patient outcomes will improve, and the whole condition will be taken care of.

## Case presentation

A 56-year-old woman reporting to the emergency department complained of 3-day painful progressive swelling on the left leg. She denied shortness of breath, fever, or chest pain.

She had no pre-existing hypertension, diabetes, chronic inflammatory disease, or malignancy. She was not on any long-term treatment with hormone supplements. No recent vigorous activity or travel history. She was fairly mobile and active.

Her vitals were stable, and examination showed the left leg >3cm more swollen than the right, no erythema, warmth, or vascular deficit. Her past medical history was significant for a recent sacral nerve stimulator implantation for bladder and bowel control. She also reported a positive family history of blood clots.

The patient's lab findings indicated several significant findings. Urine electrolytes and urea were within the normal range; sodium was 137 mmol/L, potassium 4.3 mmol/L, and creatinine 57 umol/L, indicating stable renal function with a GFR greater than 90 ml/min. But her CRP was considerably high at 158 mg/L. Hemoglobin level was 131 gL and her WBC level was 13.2 x 10^9/L, which is indicative of an inflammatory response.

A thrombophilia and vasculitis screen was performed in the outpatient (GP) setting as part of the initial work-up. This included autoimmune serology (ANA, rheumatoid factor, smooth muscle antibody, mitochondrial antibody, liver-kidney microsomal antibody, gastric parietal cell antibody, antiphospholipid antibodies including β2-glycoprotein I IgG/IgM and anticardiolipin IgG, and tissue transglutaminase IgA). It also included coagulation studies assessing antithrombin, protein C, protein S levels, and APC resistance. Lupus-sensitive APTT was also performed for anti-phospholipid syndrome assessment, as shown in Table 1, though interpretation was limited due to concurrent apixaban therapy. Mixing studies showed correction of clotting times, suggesting no circulating inhibitor. Genetic testing identified a prothrombin G20210A mutation. The thrombophilia screen had a positive heterozygous prothrombin 20210A allele, which is a genetic risk factor of venous thromboembolism. D-dimer was found to be highly increased at 10334 ng/mL as a symptom of the suspected DVT, whereas the ratio of the APTT was 1.3, and the INR was 1.2, which was in line with the anticoagulation therapy that is being administered (Table 2).

**Table 1:** The vasculitis screen This table summarizes the patient’s autoimmune and thrombophilia screening results, including serological and coagulation-based assays.

Test	Result	Reference Range
Rheumatoid Factor	<10 IU/mL	0-14 IU/mL
Antinuclear Antibody (ANA)	Negative	
Smooth Muscle Antibody	Negative	
Gastric Parietal Cell Antibody	Negative	
Mitochondrial Antibody	Negative	
Liver Kidney Microsomal Antibody	Negative	
Beta-2 Glycoprotein I Antibody (lgG)	9.8U	0-20U
Beta-2 Glycoprotein I Antibody (lgM)	14.7U	0-20U
Tissue Transglutaminase Antibody (IgA)	<1.9U	0-20U
Cardiolipin Antibody (IgG)	3.0U	0-20U
Activated Partial Thromboplastin Time (APTT) – Reagent 2	47.2s	26-36 s
Antithrombin	151 IU/dL	82-124 IU/dL
Protein C	127 IU/dL	73-153 IU/dL
Free Protein S	103.8 IU/dL	58-140 IU/dL
Activated Protein C Ratio	3	2.5-6.0
Genetic testing- Prothrombin 20210A Allele	Heterozygous	
50% Patient:50% Normal (0 hr)	44.10s	
20% Patient :80% Normal (0 hr)	38.60s	

**Table 2:** Coagulation screening of case study

Test	Result	Reference Range
International Normalised Ratio (INR)	1.2	0.9-1.1
Activated Partial Thromboplastin Time (APTT)	31.6s	22.5-28.0s
Activated Partial Thromboplastin Time Ratio (APTT Ratio)	1.3	-
Prothrombin Time (PT)	14.0s	10-13 s
D-dimer (Fibrin Degradation Product)	10,334 ng/mL	0-230 ng/mL

Table 2 summarises the patient’s coagulation profile and fibrinolytic markers. Prothrombin time (PT) and international normalized ratio (INR) assess the extrinsic and common pathways, while activated partial thromboplastin time (APTT) and APTT ratio evaluate the intrinsic and common pathways, with the ratio standardising results against a normal control. D-dimer, a fibrin degradation product, reflects fibrinolysis and is elevated in states of active thrombosis or increased clot turnover.

The Doppler ultrasound of the left lower limb veins showed occlusive thrombosis in the left common femoral vein, with the proximal end not identified. An immediate CT venography demonstrated extensive deep vein thrombosis extending from the left common iliac vein to the popliteal vein. The findings were consistent with left-sided venous outflow obstruction due to May-Thurner syndrome at the level of the left common iliac vein, with proximal extension of thrombus into the inferior vena cava. Prominent pelvic venous collaterals were also noted.

Following CT venography demonstrating extensive iliofemoral DVT, a contrast-enhanced CT abdomen and pelvis was performed to further evaluate the underlying cause of venous compression and to exclude secondary pelvic or abdominal pathology. This confirmed compression of the left common iliac vein by the right common iliac artery against the lumbar vertebrae, in keeping with May-Thurner syndrome, with associated extensive left iliofemoral DVT.

The vascular team was involved, who advised against any immediate intervention, recommended 6-12 months of anticoagulation, and discussed the case with the multidisciplinary team. She was then discharged on Apixaban along with a venous thromboembolism clinic follow-up.

**Figure 1:**
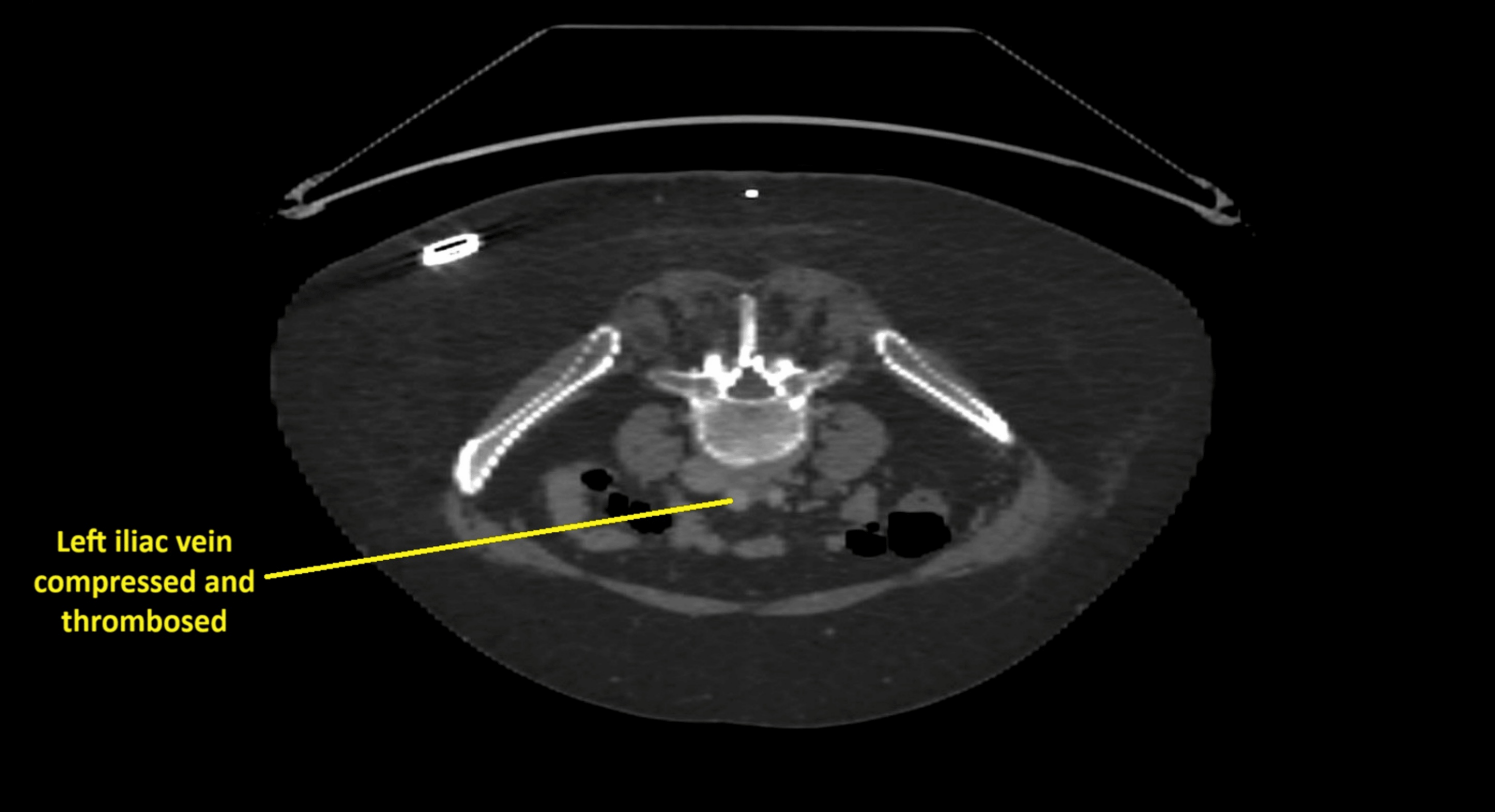
CT with contrast of the abdomen at the transverse section showing the left iliac vein compressed and thrombosed

**Figure 2:**
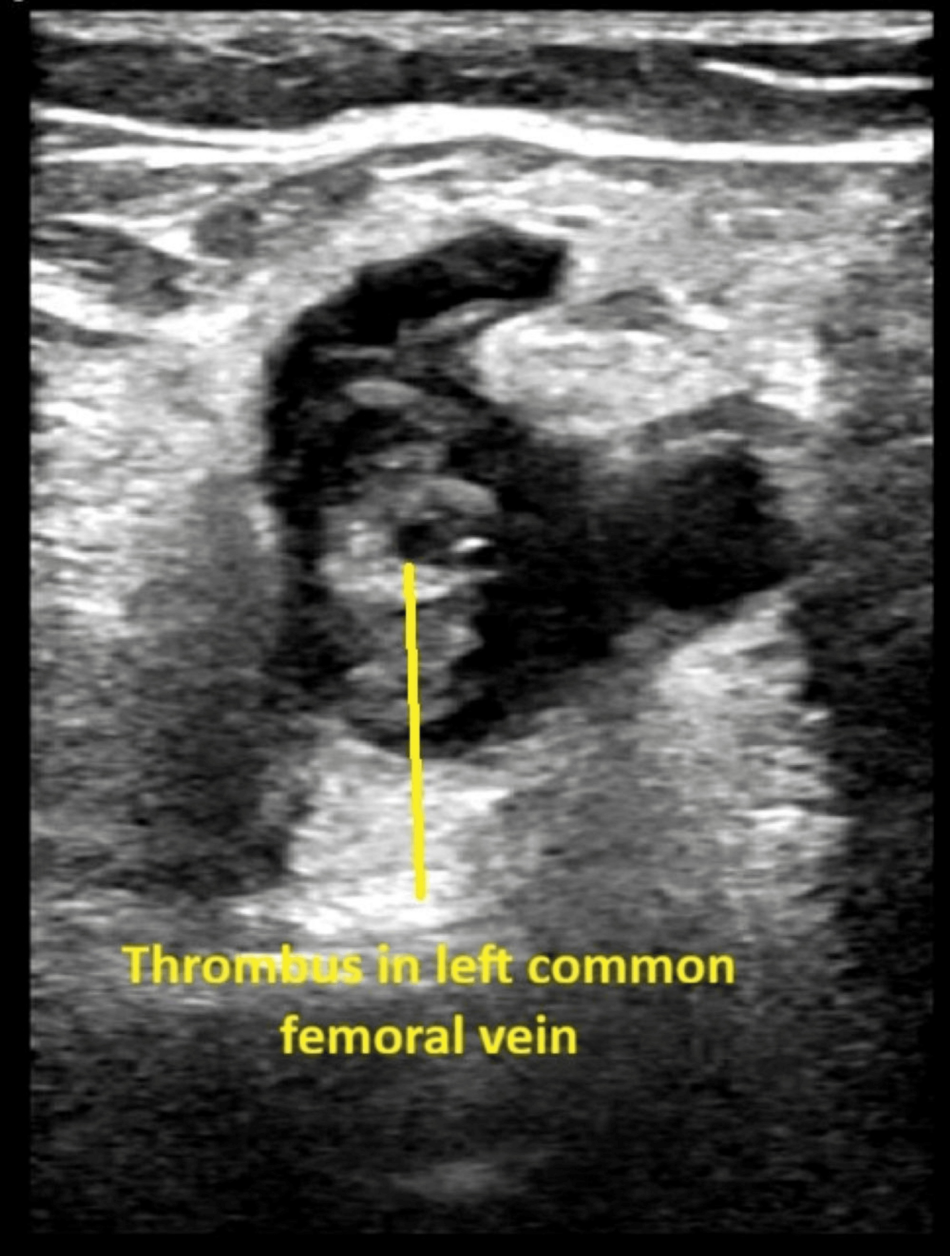
Enlarged section of the CT image showing thrombus in the left common femoral vein

**Figure 3:**
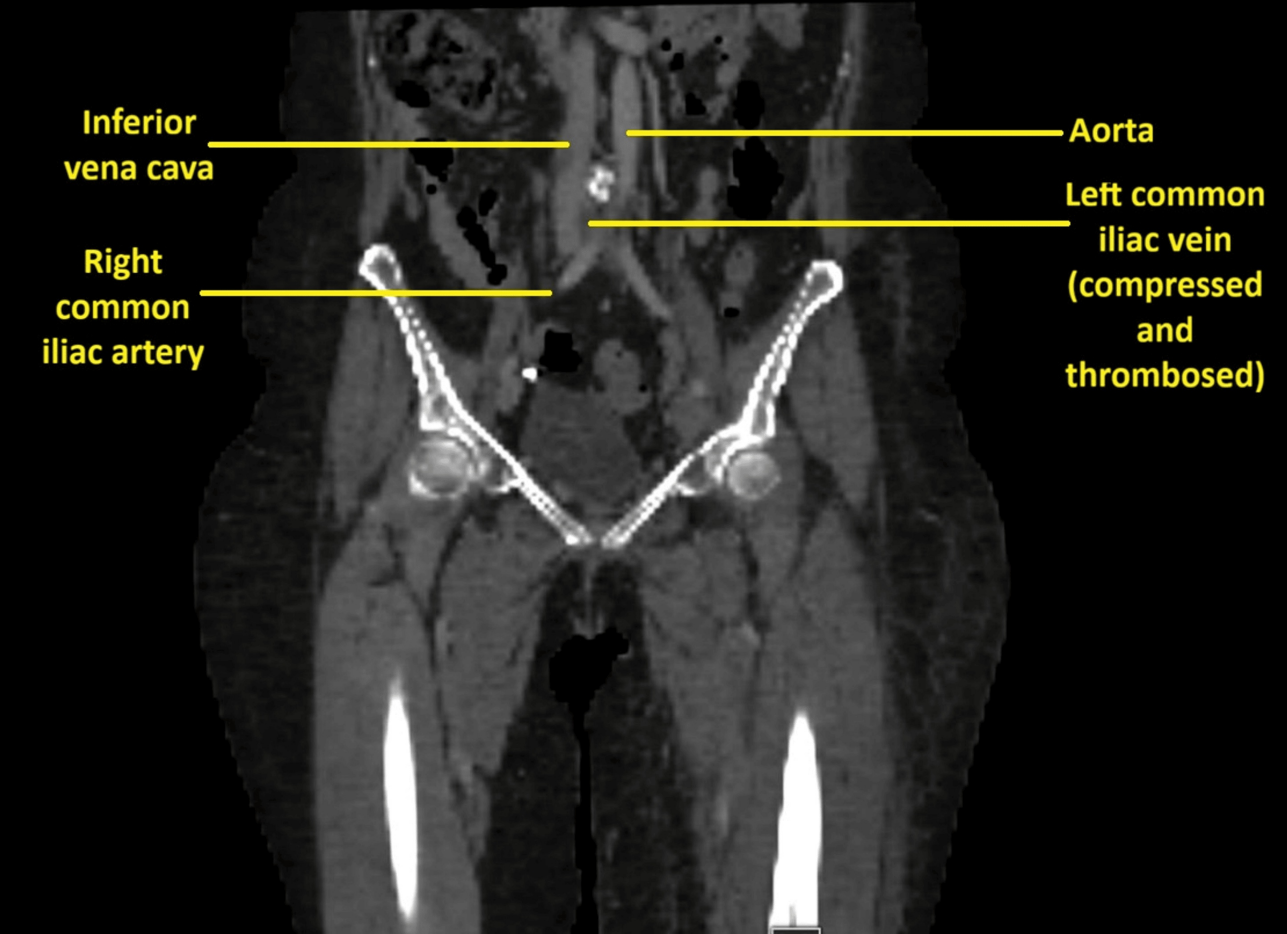
Sagittal section of abdomen and pelvis showing the left common vein compressed and thrombosed

Due to worsening leg swelling and pain, a repeat CT abdomen with portal venous phase contrast was performed to visualise the extension of the clot (Figures 1-3). Comparison with the previous CT showed a persistent thrombus within the left iliac vein and a partially imaged left femoral vein, with a significant reduction in thrombus volume, as evidenced by a reduction in the vein's calibres.

The patient's condition was managed symptomatically without the need for surgical intervention, and she was advised to use class 2 compression stockings, analgesics, and anticoagulants.

## Discussion

Compression of the left common iliac vein by the right common iliac artery leads to the development of May-Thurner Syndrome (MTS), a prevalent factor contributing to venous irregularities in the left lower limb [1]. The left common iliac vein experiences partial obstruction due to the overlying artery, which physically constrains it, and prolonged pulsatile stresses lead to significant intimal hypertrophy [1]. MTS is estimated to occur in 2%-5% of individuals evaluated for conditions related to the veins of the lower limbs [2]. The literature review indicates that the occurrence of deep venous thrombosis (DVT) in the left lower extremity is significantly higher, being three to eight times more prevalent than on the right side [3,4]. Identifying iliac vein compression is essential to prevent the occurrence of iliofemoral venous thrombosis and venous insufficiency syndrome. Iliac venography via the femoral approach remains the most recognized diagnostic technique, offering direct visualization of compression and the capability to measure the pressure gradient to assess hemodynamic significance. Contrast-enhanced CT images of the distal abdomen reveal extrinsic compression by the right common iliac artery. Chronic May-Thurner syndrome has the potential to lead to enduring impairment [5]. Possible sequelae include leg oedema, varicosities, DVT, chronic venous insufficiency, and complications such as pulmonary embolism or phlegmasia cerulea dolens in cases of complete occlusion [1,6,7]. Various surgical techniques are available, such as vein-patch angioplasty, which involves the removal of intraluminal bands, the division and displacement of the right common iliac artery behind the left common iliac vein or inferior vena cava, and bypass of the ipsilateral common femoral vein with creation of a temporary arteriovenous fistula [8]. The long-term success rate, characterized by the patency of the left common iliac vein or venous bypass, ranges from 74% to 80% [9,10]. Recently, endovascular procedures, especially the implantation of stents, have shown impressive outcomes in both the short and medium term [11,12].

In the present case, the initial Doppler ultrasound failed to show the proximal extent of the thrombus, highlighting a limitation of ultrasound in assessing the iliac veins due to bowel gas overlap and the deep positioning of these vessels within the pelvis. Subsequent CT venography (CTV) provided conclusive results [13-15]. This aligns with other researchers who have indicated that CTV provides the anatomical clarity needed to distinguish between bland DVT and MTS by identifying the point of iliac vein compression relative to the lumbar vertebrae [16]. 

The approach of treating our patient with Apixaban alone represents a cautious treatment strategy, given the established efficacy and safety profile of direct oral anticoagulants (DOACs) [17]. Sawafta et al. observed that while anticoagulation can halt the further development of clots, the existing mechanical compression remains, leading to frequent recurrence of DVT unless the obstruction is addressed, often necessitating lifelong anticoagulation in such scenarios [18]. Conversely, subsequent sub-analyses revealed that patients presenting with high proximal DVT, such as our patient with iliofemoral involvement, may require more aggressive initial intervention to prevent the onset of post-thrombotic syndrome (PTS) [19,20]. 

Nevertheless, the decision by the vascular unit to defer definitive intervention, prioritizing clinical stability and outpatient multidisciplinary team (MDT) consultation, can be supported by contemporary evidence. Jeon et al. suggest that prolonged anticoagulation may facilitate the development of sufficient collateral circulation in individuals with MTS, as illustrated by our patient's CT images showing pelvic varicose collaterals [21]. This suggests a potential compensatory process that might obviate the need for immediate stenting in certain clinical scenarios. 

In response to the limitations of anticoagulation alone in MTS, as highlighted by Dwivedi et al., the recommended alternative is typically catheter-directed thrombolysis (CDT) [22]. Additionally, a review by Avgerinos et al. has validated that common iliac vein stenting outperforms anticoagulation alone [23]. The significant burden of thrombus in the common, external, and femoral veins often makes a patient eligible for these procedures. A recent investigation contributed further to this discussion, revealing that pharmacomechanical catheter-directed thrombolysis did not correlate with a reduced risk of post-thrombotic syndrome in patients with iliofemoral DVT but was associated with a reduction in symptom severity [20]. Given these findings, the surveillance and MDT approach implemented for our patient may be deemed acceptable as long as her symptoms are managed effectively. 

The patient was successfully initiated on Apixaban therapy. A study by Miceli et al. discusses the increasing acceptance of DOACs in the management of MTS, highlighting that they offer a more favorable safety profile compared to warfarin [24]. 

The recent implantation of a sacral nerve stimulator in this patient likely contributed to the exacerbation of a pre-existing anatomical compression. Left common iliac vein compression is often asymptomatic until a secondary event, such as surgery, trauma, or immobilization, precipitates a thrombotic incident [13,25]. This finding in our case indicates that a background of perioperative inflammation, coupled with a family history of thrombosis and an undiagnosed thrombophilia (heterozygous prothrombin 20210A mutation), may overwhelm the venous hemodynamic reserve of a patient with underlying MTS.

Identifying iliac vein compression is essential to prevent the occurrence of iliofemoral venous thrombosis and venous insufficiency syndrome. Iliac venography via the femoral approach remains the most recognized diagnostic technique, offering direct visualization of compression and the capability to measure the pressure gradient to assess hemodynamic significance. Contrast-enhanced CT images of the distal abdomen reveal extrinsic compression by the right common iliac artery. Chronic May-Thurner syndrome has the potential to lead to enduring impairment [5]. Possible sequelae include leg edema, varicosities, DVT, chronic venous insufficiency, and more severe issues such as pulmonary embolism or phlegmasia cerulea dolens in cases of complete occlusion [1,6,7].

Various surgical techniques are available, such as vein-patch angioplasty, which involves the removal of intraluminal bands, the division and displacement of the right common iliac artery behind the left common iliac vein or inferior vena cava, and bypass of the ipsilateral common femoral vein with creation of a temporary arteriovenous fistula [8]. The long-term success rate, characterized by the patency of the left common iliac vein or venous bypass, ranges from 74% to 80% [9,10]. Recently, endovascular procedures, especially the implantation of stents, have shown impressive outcomes in both the short and medium term [11,12].

In the present case, the initial Doppler ultrasound failed to show the proximal extent of the thrombus, highlighting a limitation of ultrasound in assessing the iliac veins due to bowel gas overlap and the deep positioning of these vessels within the pelvis. Subsequent CT venography (CTV) provided conclusive results [13-15]. This aligns with other researchers who have indicated that CTV provides the anatomical clarity needed to distinguish between bland DVT and MTS by identifying the point of iliac vein compression relative to the lumbar vertebrae [16]. 

The approach of treating our patient with Apixaban alone for 6-12 months represents a cautious treatment strategy, given the established efficacy and safety profile of direct oral anticoagulants (DOACs) [17]. Sawafta et al. observed that while anticoagulation can halt the further development of clots, the existing mechanical compression remains, leading to frequent recurrence of DVT unless the obstruction is addressed, often necessitating lifelong anticoagulation in such scenarios [18]. Conversely, subsequent sub-analyses revealed that patients presenting with high proximal DVT, such as our patient with iliofemoral involvement, may require more aggressive initial intervention to prevent the onset of post-thrombotic syndrome (PTS) [19,20].

Nevertheless, the decision by the vascular unit to defer definitive intervention, prioritizing clinical stability and outpatient multidisciplinary team (MDT) consultation, can be supported by contemporary evidence. Jeon et al. suggest that prolonged anticoagulation may facilitate the development of sufficient collateral circulation in individuals with MTS, as illustrated by our patient's CT images showing pelvic varicose collaterals [21]. This suggests a potential compensatory process that might obviate the need for immediate stenting in certain clinical scenarios. 

In response to the limitations of anticoagulation alone in MTS, as highlighted by Dwivedi et al., the recommended alternative is typically catheter-directed thrombolysis (CDT) [22]. Additionally, a review by Avgerinos et al. has validated that common iliac vein stenting outperforms anticoagulation alone [23]. The significant burden of thrombus in the common, external, and femoral veins often makes a patient eligible for these procedures. A recent investigation contributed further to this discussion, revealing that pharmacomechanical catheter-directed thrombolysis did not correlate with a reduced risk of post-thrombotic syndrome in patients with iliofemoral DVT but was associated with a reduction in symptom severity [20]. Given these findings, the surveillance and MDT approach implemented for our patient may be deemed acceptable as long as her symptoms are managed effectively. 

The patient was successfully initiated on Apixaban therapy. A study by Miceli et al. discusses the increasing acceptance of DOACs in the management of MTS, highlighting that they offer a more favorable safety profile compared to warfarin [24]. 

The recent implantation of a sacral nerve stimulator in this patient likely contributed to the exacerbation of a pre-existing anatomical compression. Left common iliac vein compression is often asymptomatic, and individuals remain stable until a secondary event, such as surgery, trauma, or immobilization, precipitates a thrombotic incident [13,25]. This finding in our case indicates that a background of perioperative inflammation, coupled with a family history of thrombosis and an undiagnosed thrombophilia (heterozygous prothrombin 20210A mutation), may overwhelm the venous hemodynamic reserve of a patient with underlying MTS.

**Figure 4:**
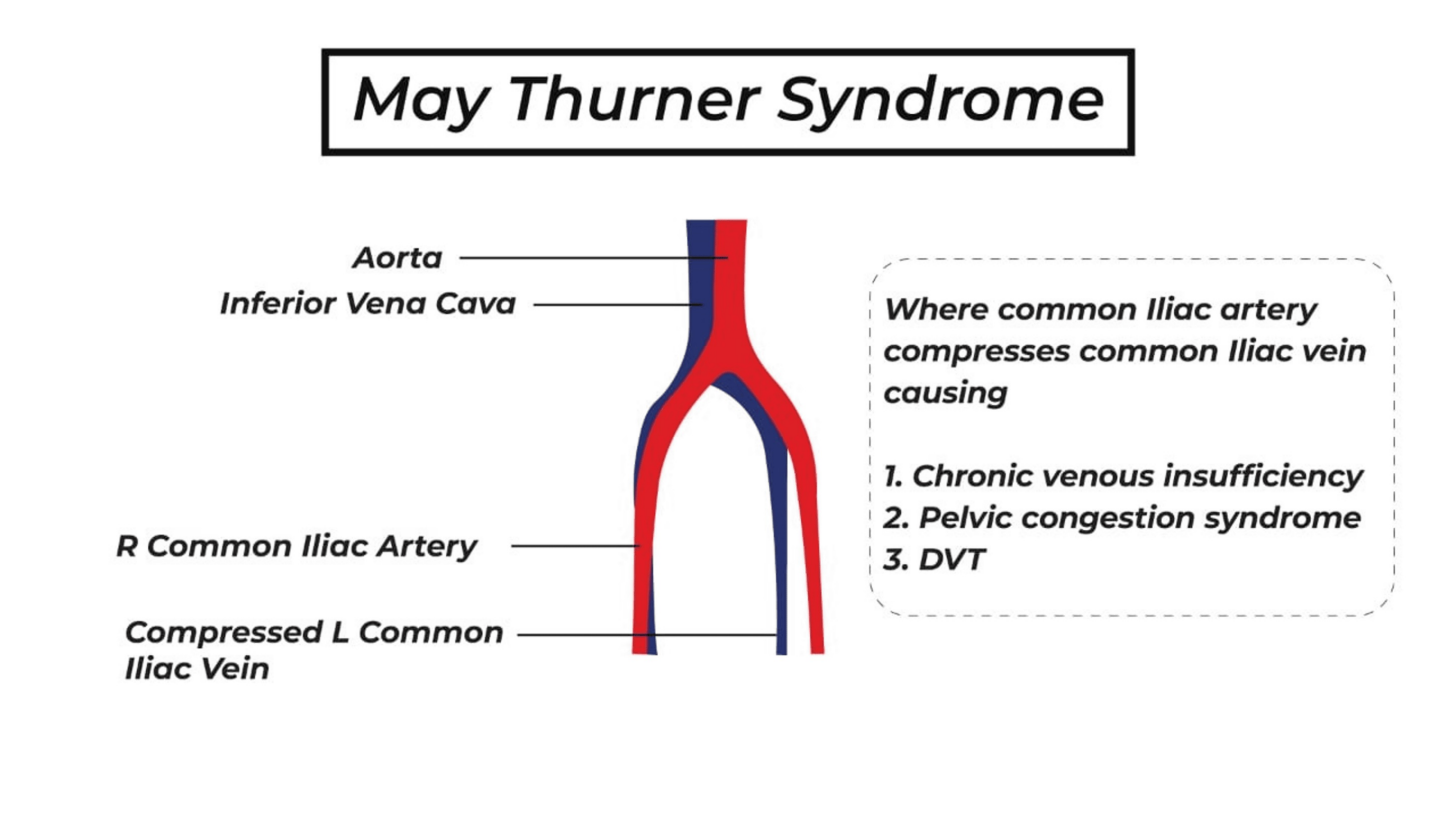
Anatomy of May-Thurner syndrome This image has been created by Illustrator.

Right-sided May-Thurner syndrome is rare and usually reflects a May-Thurner-like physiology rather than the classic entity. It may result from anatomical vascular variants (abnormal crossing or course of iliac vessels), or secondary external compression by pelvic or retroperitoneal masses, lymphadenopathy, pregnancy, or post-surgical/structural changes, but needs more literature analysis. Figure 4 shows the anatomy of May-Thurner syndrome.

## Conclusions

The case demonstrates that it is necessary to discuss May-Thurner syndrome in the presentation of iliofemoral left DVT with the help of the identification of risk factors among the patient, including a history of surgery or a predisposition to this syndrome in a family. An early imaging method and a teamwork approach to care should be used to manage the conservative care of the condition. Anticoagulation is the cornerstone of the management process, and close monitoring is necessary in order to prevent recurrence in patients.

Any femoral DVT where the proximal end is not identified should be followed immediately by further imaging to rule out clot size and uncommon causes of unprovoked DVT, as May-Thurner Syndrome. May-Thurner syndrome should also be suspected in chronic left-sided venous insufficiency in adults, particularly ladies aged 20 to 50. Right-sided May-Thurner syndrome is rare and usually reflects a May-Thurner-like physiology rather than the classic entity. It may result from anatomical vascular variants (abnormal crossing or course of iliac vessels), or secondary external compression by pelvic or retroperitoneal masses, lymphadenopathy, pregnancy, or post-surgical/structural changes, but needs more literature analysis.

## References

[1] Obrand DI, Mijangos J, Brassard R (1998). Ureteropelvic urinary extravasation due to iliac artery aneurysm. Ann Vasc Surg.

[2] Hng J, Su S, Atkinson N (2021). May-Thurner syndrome, a diagnosis to consider in young males with no risk factors: a case report and review of the literature. Jr Med Cas Rep.

[3] Gong S, Sung Kim J, Kim H (2020). Effect of laterality and location of deep vein thrombosis on pulmonary embolism occurrence. Clin Surg.

[4] Abdelmalik BHA, Leslom MMA, Gameraddin M (2023). Assessment of lower limb deep vein thrombosis: characterization and associated risk factors using triplex doppler imaging. Vasc Health Risk Manag.

[5] Montes MC, Carbonell JP, Gómez-Mesa JE (2021). Endovascular and medical therapy of May-Thurner syndrome: Case series and scoping literature review. J Med Vasc.

[6] Shah M, Vayzband V, Nabi M, Chandna S (2022). Phlegmasia cerulea Dolens due to May Thurner syndrome. Brown J Hosp Med.

[7] Serrano Reyes JC, Pinilla R, Chanis G (2025). Mechanical thrombectomy for iliofemoral deep venous thrombosis complicated by Phlegmasia cerulea Dolens in a pregnant patient with May-Thurner syndrome: a case Report. Cureus.

[8] Alrabadi B, Al Kayed HA, Alshujaieh N (2024). Impact of beta-blocker therapy on pregnant women with long QT syndrome: a systematic review. Cureus.

[9] Kölbel T, Lindh M, Akesson M (2009). Chronic iliac vein occlusion: midterm results of endovascular recanalization. J Endovasc Ther.

[10] Greenberg JI, May S, Suliman A, Angle N (2008). The brachial artery-brachial vein fistula: expanding the possibilities for autogenous fistulae. J Vasc Surg.

[11] Nguyen D, Berman SS, Balderman JA (2024). Initial experience with the ambulatory management of acute iliofemoral deep vein thrombosis with May-Thurner syndrome with percutaneous mechanical thrombectomy, angioplasty and stenting. J Vasc Surg Venous Lymphat Disord.

[12] Ali MM, Hasan SA, Qaheri RS (2023). Endovascular stenting for May-Thurner syndrome: a case report. Cureus.

[13] Brinegar KN, Sheth RA, Khademhosseini A (2015). Iliac vein compression syndrome: Clinical, imaging and pathologic findings. World J Radiol.

[14] Eliahou R, Sosna J, Bloom AI (2012). Between a rock and a hard place: clinical and imaging features of vascular compression syndromes. Radiographics.

[15] Mousa AY, AbuRahma AF (2013). May-Thurner syndrome: update and review. Ann Vasc Surg.

[16] Maroun A, Quinn S, Dushfunian D (2023). Clinical applications of four-dimensional flow MRI. Magn Reson Imaging Clin N Am.

[17] Franchini M, Mannucci PM (2016). Direct oral anticoagulants and venous thromboembolism. Eur Respir Rev.

[18] Malik M, Naveed MA, Asif MA (2025). B-cell lymphoblastic lymphoma patient presenting with life threatening ruptured mycotic aneurysm of internal iliac artery. Radiol Case Rep.

[19] Cohen AT, Agnelli G, Anderson FA (2007). Venous thromboembolism (VTE) in Europe. The number of VTE events and associated morbidity and mortality. Thromb Haemost.

[20] Vedantham S, Goldhaber SZ, Julian JA (2017). Pharmacomechanical catheter-directed thrombolysis for deep-vein thrombosis. N Engl J Med.

[21] Wienbeck S, Fischbach R, Kloska SP (2010). Prospective study of access site complications of automated contrast injection with peripheral venous access in MDCT. AJR Am J Roentgenol.

[22] Bhargava T, Yadav M, Vijayavargiya N (2024). Evaluating the effect of NanoFilled composite restorations on the wear resistance of posterior teeth: an RCT. J Pharm Bioallied Sci.

[23] Avgerinos ED, Black S, van Rijn MJ, Jalaie H (2024). The role and principles of stenting in acute iliofemoral venous thrombosis. J Vasc Surg Venous Lymphat Disord.

[24] Rodríguez-Fernández S, Egido-Moreno S, Rodríguez-Fernández S (2025). Association between oral lichen planus and thyroid disease: a cross-sectional study. J Clin Med.

[25] Wu CY, Li CF, Wu QJ (2017). Chinese systemic lupus erythematosus treatment and research group registry IX: clinical features and survival of childhood-onset systemic lupus erythematosus in China. Chin Med J (Engl).

